# Factors influencing the choice of specialization - a cross-sectional study with civilian medical students and prospective medical officers in Germany

**DOI:** 10.1186/s12909-024-06173-9

**Published:** 2024-10-17

**Authors:** Katja Goetz, Katharina Grienitz, Jost Steinhäuser

**Affiliations:** Institute of Family Medicine, University Medical Center Lübeck, 23562 Lübeck, Schleswig-Holstein Germany

**Keywords:** Career planning, Medical care, Study place allocation, Choice of specialty, Physician shortage

## Abstract

**Background:**

The recruitment of physicians is an increasing challenge. The aim of the study was to explore factors that might influence future civilian and military doctors’ choice of specialization.

**Methods:**

A 28 item author developed questionnaire was used to survey civilian medical students and prospective medical officers. The questionnaire consisted of 20 items addressing choice of specialization, and eight socio-demographic factors. Response options were available in the form of open and closed questions as well as in the form of a Likert scale (1 ‘not at all’ to 6 ‘very much’). The questionnaire was completed online by prospective medical officer from June to September 2021 and by civilian students from November 2020 to March 2021.

**Results:**

In total, 2,030 students (56.1% female, mean age = 24, mean semester 6.5) participated in the survey, 1,553 civilian students and 477 prospective medical officers. Regular feedback and clear structures (mean = 5.35) followed by good teamwork (mean = 5.33) during postgraduate training was rated particularly important. Moreover, a secure job after graduation (mean = 5.23) and a compatibility of family and job (mean = 5.14) were important parameters for career choice. The specializations most frequently chosen were surgery (32.0%), internal medicine (27.1%) and anesthesiology (22.1%). The regression models showed that the choice depends on own attitude concerning the specialty would be a patient-orientation job, or a job with more manual work or more career option within in the job preferred.

**Conclusions:**

Considering the value students place on receiving feedback and the structure of their specialist training, this seems to be a promising strategy for future recruitment. Good team dynamics and job security are key concerns for prospective physicians.

**Supplementary Information:**

The online version contains supplementary material available at 10.1186/s12909-024-06173-9.

## Background

There is a worldwide increasing demand for medical professionals. Although the total number of doctors has increased in recent years in Germany, a decline in the time spent working in the health sector is noticeable [1]. The situation is exacerbated by changes in the distribution of physicians, by changes in the allocation of study places as well by the turnover of medical staff [2].

Generally, strategies against physician shortage may be divided into three categories: normative, utilitarian, and coercive measures [3]. In Germany these strategies are used as follows: Utilitarian measures are offered most frequently. These are basically financial incentives. For example, a €60,000 bonus as an incentive is offered for taking up practice in an underserved rural area [4].

The normative approach describes interventions which occur at the education and training stages [3]. An example of this type of intervention is the optimization of the postgraduate training of General Practice trainees through educational meetings, mentoring scheme and a Train the Trainer course [5].

The stipulation that doctors cannot open up a practice in certain well-staffed regions may be considered a coercive measure [3, 6]. Hybrid strategies may include all three strategy-types, e.g. studying by the Military Armed Forces or conditional university offers and scholarships. Applicants are accepted on the condition that they commit to a certain place of practice and/or time period [7–9].

There are no tuition fees to be paid in Germany for studying medicine. Civilian and military students’ study at the same universities. However, there are differences regarding the access to medicine school: The German Armed Forces is a volunteer army. Due to the suspension of compulsory military service in 2011, the German military must also compete with civilian employers [10, 11]. Studying human medicine in the German Armed Forces entails a 17-year commitment period. Prior to enrolment, applicants attend an assessment center which over several days checks their suitability to study and serve as an officer. In the selection process for studying medicine in the German Armed Forces, pre-university grade point average (pu-GPA) play a lesser role than in the standard application process for medical studies at an university, in that other personal factors are taken into account. For example, applicants must complete computer tests on mathematical and technical thinking, a fitness test, a group problem-solving task and tests specific to the course of study [12, 13].

At the beginning of the commitment period, candidates complete a three-month basic military training. Following this, the prospective medical officers begin their studies at a civilian university. During the period of study, clerkships in military facilities, an officer training course and regular fitness training must be completed. After the six-year course of study, the medical officers start their first clinical assignment at one of the five military hospitals in Germany and then go on to one of many non-hospital assignments. A focus during postgraduate training is in emergency medicine [12, 14].

As is the case with civilian students, prospective medical officers are able to select a specialization at the end of their undergraduate studies. However, unlike in the civilian system, the choice of specializations and the hospital is determined by a points system. The points system is comprised of study credits and additional study-related credits [15].

For civilian students the medical school admission requirements the pu-GPA are of greatest importance within different quotas: 30% are chosen solely based on the pu-GPA, 60% are chosen by the different university-specific selection procedures in which two pu-GPA-independent selection criteria have to be considered and only 10% of the study places are assigned based solely on pu-GPA-independent criteria [16]. Therefore, there are two quite different chosen groups of students within the University system in Germany offering the chance to survey, whether factors influencing students in their choice of specialization differ, depending on the way to medicine school.

Understanding the factors which influence students in their choice of specialization is important in order to meet demands of the students and adjust selection criteria if necessary. Therefore, the aim of the study was to explore factors that might influence future civilian and military doctors’ choice of specialization.

## Methods

A cross-sectional study design was used to evaluate factors that might influence choice of specialization measured by a self-developed questionnaire. The study was complied with the STROBE guidelines (Strengthening the Reporting of Observational Studies in Epidemiology) [17] [see additional file S1].

### Questionnaire

The questionnaire was created based on a literature search using the search terms [“medical studies in the German military”, “prospective medical officer”, “choice of area”, “clinical traineeship”, “nursing traineeship”, “specialist training”] and also drew on the experience of two of the authors (KGr medical officer and JS, general practitioner and expert in postgraduate training research). It was divided into two sections. Part one contained questions about students’ skills and abilities and part two questions about general parameters of medical practice. There were 20 questions in total. For this purpose, 6-point Likert scales (1 = not important / relevant at all, 6 = very important/ relevant) were used.

Prior to being distributed, the questionnaire was evaluated using the concurrent think-aloud method by two medical students [18]. As a result of this evaluation, some questions were replaced, supplemented, or reworded. The online questionnaire was filled in anonymously using SurveyMonkey©. The questionnaire can be found in the additional material [see additional file S2].

### Recruitment

Civilian students were contacted about the 38 student councils in Germany with the request to invite students to participate in an online-survey between November 2020 and March 2021 (with two reminders at four-week intervals). After the period of formal testing, the German Armed Force supported the recruitment. The survey was sent to all prospective medical officers by email in the period between June 2021 and September 2021 (with also two reminders at four-week intervals).

### Statistical analysis

A statistical analysis was carried out using IBM SPSS version 27.0. First, the psychometric properties of the questionnaire in terms of internal consistency reliability and factorial validity were determined. Exploratory factor analysis with extraction of factor loadings were performed to test the dimensionality of the individual variables for the topic “student skills and abilities” and “general parameters of medical practice”. The component loadings were subjected to Varimax rotation, and their number was determined by eigenvalues > 1. Furthermore, sample suitability was evaluated with the Kaiser-Meyer-Olkin (KMO) criterion, and Bartlett’s test was performed to examine sphericity (*p* < 0.05) [19]. All items with a component loading > 0.4 were assigned to the particular factor. Internal consistency was determined by Cronbach’s α [20]. Values > 0.8 represent good internal consistency, while values > 0.6 and values > 0.4 represent acceptable consistency and poor internal consistency, respectively.

Second, a descriptive analysis of the data was performed. The test for socio-demographic differences between the four most frequently mentioned specializations was carried out for metric data are using the students t-test for mean differences and for categorical data are using the chi-square test. The different factors which might influence the choice of occupation were compared between civilian and military students by using students’ t-test. Furthermore, the calculation of linear correlations between the various specializations, the socio-demographic variables and the various influencing factors was carried out using Pearson’s correlation coefficient. The variables were left exclusively in their original coding for this purpose. Testing for connections using a correlation is a common procedure to reduce the number of variables for subsequent model calculations. In a further step, significant correlations identified in this way were considered in a binary-logistic regression with the dependent categorized variable (desired specialization). The four different disciplines resulted in four regression models. To test for goodness of fit for logistic regression models, the Hosmer-Lemeshow test was considered [21]. The evaluation of the size of the effects from the regression models were determined by interpreting the standardized beta (β) coefficient. Values > 0.5 represent strong effect, while values between 0.2 and 0.5 moderate and < 0.2 weak effect [22]. An alpha level *p* ≤ 0.05 was used for tests of statistical significance.

### Ethics

Participants gave informed consent to taking part in the study following an online information session about the study. In this session, participants’ attention was drawn to the content and benefits of the study, and they were made aware of data protection information. On 12 November 2020, the ethics committee of the University of Lübeck approved the ethics application under file number 20–354.

## Results

A total of *n* = 2,030 medical students took part in the online survey.

### Psychometric properties

For the topic “students’ skills and abilities” the exploratory factor analysis revealed a two-factor solution with a total variance (R²) of 65% (KMO = 0.68, Bartlett’s test for sphericity *P* < 0.001) [please see in detail additional file S3]. The Cronbach’s α value for internal consistency was 0.66. For the topic “general parameters of medical practice” the exploratory factor analysis revealed a five-factor solution with a R² of 56.3% (KMO = 0.68, Bartlett’s test for sphericity *P* < 0.001) [please see in detail additional file S3]. The Cronbach’s α value for internal consistency was 0.58. The variable “importance of practicing a specialization which requires manual work” received no clear factor loading. After leaving this item from the topic the Cronbach’s α value for internal consistency was 0.60.

### Socio-demographics

More women than men responded to the questionnaire. The mean age was 23.9 years (SD = 3.8). Details of the socio-demographics also divided for the two groups - civilian students and prospective medical officers -are shown in Table 1.

**Table 1:** Socio-demographics of the participants

Variables*		All students (*n* = 2,030), mean (SD)	Civilian students (*n* = 1,553), mean (SD)	Prospective medical officers (*n* = 477), mean (SD)
Age in years		23.9 (3.8)	24.1 (4.1)	23.5 (3.0)
Semester		6.5 (3.1)	6.4 (3.2)	6.5 (3.1)
Variables*		**Number (%)**	**Number (%)**	**Number (%)**
Sex	Male	537 (26.5)	346 (22.3)	191 (40.0)
	Female	1,138 (56.1)	943 (60.7)	195 (40.0)
	Divers	10 (0.5)	7 (0.5)	3 (0.6)
Have children		78 (3.8)	67 (4.3)	11 (2.3)
In a committed relationship		734 (36.2)	574 (37.0)	160 (33.5)
preferred working hours	40 h or less	943 (46.5)	750 (48.3)	193 (40.5)
	Up to 60 h	747 (36.8)	550 (35.4)	197 (41.3)
Target position	Head physician in hospital	132 (6.5)	94 (6.1)	38 (8.0)
	Senior physician in hospital	538 (26.5)	393 (25.3)	145 (30.4)
	Specialist in hospital	195 (9.6)	147 (9.5)	48 (10.1)
	Independent specialist in private practice	542 (26.7)	433 (27.9)	109 (22.9)
	Employed specialist in private practice	159 (7.8)	127 (8.2)	32 (6.7)

* n varies due to missing data; SD: Standard deviation

Overall, 35 from 38 university locations were included in the survey. Moreover, most of the participants were located at Berlin and Lübeck. Details are shown in Table 2.

**Table 2:** Overview of the participating university locations (*n* = 2,030)

Location	Number (%)
Berlin	390 (19.2)
Lübeck	251 (12.4)
Köln	132 (6.5)
Bochum	90 (4.4)
Ulm	86 (4.2)
Tübingen	74 (3.6)
München	72 (3.5)
Göttingen	61 (3.0)
Mainz	61 (3.0)
Hamburg	57 (2.8)
Greifswald	49 (2.4)
Regensburg	49 (2.4)
Bonn	38 (1.9)
Heidelberg	37 (1.8)
Aachen	21 (1.0)
Frankfurt/ Main	19 (0.9)
Leipzig	18 (0.9)
Dresden	16 (0.8)
Rostock	13 (0.6)
Jena	12 (0.6)
Saarland/ Homburg	12 (0.6)
Münster	11 (0.5)
Würzburg	11 (0.5)
Augsburg	10 (0.5)
Locations with lower of 10 participating numbers of students*	42 (1.8)
Missing	398 (19.6)

* Included: Erlangen-Nurnberg, Halle, Duisburg-Essen, Mannheim, Hannover, Magdeburg, Freiburg, Marburg, Düsseldorf, Gießen, Kiel

### Selection of specialist training

63.1% (*n* = 1,281) of the participants intended to pursue further specialist training, 35.9% (*n* = 72) of the participants don’t know yet which one’s further specialist training will be chosen and 1.0% (*n* = 21) decided to choose no further specialist training. The most frequently chosen specializations are shown in Table 3. These were surgery, anesthesiology, general practice and internal medicine. Medical students who were younger and in an earlier semester chose significantly rather surgery. Significantly more male than female students selected the specialization of anesthesiology. For the choice of general practice significant differences were shown for the age of students and sex. Elderly students and female students chosen rather general practice.

**Table 3:** Overview of the four most frequently chosen specialist fields (*n* = 748)

Variables*		Surgery (*n* = 239)	Anesthesiology (*n* = 165)	General practice (*n* = 141)	Internal medicine (*n* = 203)
Sex, n (%)	male	66 (32.7)	81 (55.9)	42 (30.9)	92 (52.0)
	female	135 (66.8)	63 (43.4)	94 (69.1)	82 (46.3)
Age in years, mean (SD)		23.5 (3.6)	25.0 (3.8)	25.2 (5.0)	24.4 (3.7)
Semester, mean (SD)		6.4 (3.2)	6.4 (3.3)	7.1 (3.1)	7.2 (2.9)
Group assignment, n (%)	Civilian students	141 (59.0)	94 (57.0)	86 (61.0)	162 (79.8)
	Prospective medical officers	98 (41.0)	71 (43.0)	55 (39.0)	41 (20.2)

* n varies due to missing data; SD: Standard deviation

### Factors influencing career choice

Concerning students’ skills and abilities medical students felt confident in their communication skills (mean = 4.92, SD 0.88), in dealing with performance pressure e.g. in emergency situations (mean = 4.56, SD 1.02), and in leadership tasks (mean = 4.30, SD 1.17), see Table 4.

Moreover, for the different parameters of medical practice it was observed that the most important reasons which influenced the choice were “to have a clear structure and regular feedback during specialist training” (mean = 5.35, SD 0.84) and “the importance of teamwork” (mean = 5.33, SD 0.91). Comparing civilian and prospective medical officers, significant differences were found which are presented within the table.

**Table 4:** Factors influencing the choice of specialty – descriptive analyses and group comparison

Variable overview*	All students (*n* = 2,030), mean (SD)	Civilian students (*n* = 1,553), mean (SD)	Prospective medical officers (*n* = 477), mean (SD)	*p*-value
Students’ skills and abilities:				
How competent do you consider yourself in terms of your communication skills?	4.92 (0.88)	**4.88 (0.88)**	**5.05 (0.86)**	**< 0.01**
How well do you think you can handle pressure to perform (e.g. in emergency situations)?	4.56 (1.02)	**4.47 (1.04)**	**4.86 (0.89)**	**< 0.01**
How well qualified do you feel for leadership tasks (e.g. leading a team)?	4.30 (1.17)	**4.20 (1.18)**	**4.62 (1.09)**	**< 0.01**
How confident do you feel about examining the musculoskeletal system?	3.37 (1.29)	**3.27 (1.27)**	**3.73 (1.27)**	**< 0.01**
How would you rate the extent of your curricular training on musculoskeletal examination?	3.05 (1.18)	3.06 (1.16)	3.00 (1.25)	0.33
**General parameters of medical practice**:				
How important is specialist training with a clear structure and regular feedback for you?	5.35 (0.84)	5.34 (0.84)	5.39 (0.84)	0.37
How important is teamwork for you?	5.33 (0.91)	5.35 (0.89)	5.28 (0.94)	0.18
How important is a secure job for you after graduation?	5.23 (0.92)	5.23 (0.89)	5.24 (1.02)	0.80
How important are learning opportunities for you?	5.12 (0.95)	**5.10 (0.96)**	**5.20 (0.92)**	**0.05**
How important is direct patient contact in your future job for you?	5.07 (1.16)	5.10 (1.15)	5.04 (1.18)	0.55
How important is the compatibility of family and job for you?	5.14 (1.14)	**5.21 (1.08)**	**4.89 (1.27)**	**< 0.01**
How important are career opportunities for you?	4.41 (1.23)	**4.37 (1.24)**	**4.56 (1.19)**	**< 0.01**
How important is it for you to achieve your specialist title as quickly as possible?	3.97 (1.36)	**3.83 (1.33)**	**4.42 (1.35)**	**< 0.01**
How important is place of residence for you when choosing a specialty?	4.54 (1.34)	**4.62 (1.28)**	**4.28 (1.49)**	**< 0.01**
How important is an option of changing specialization if necessary for you?	4.22 (1.22)	**4.25 (1.19)**	**4.12 (1.29)**	**< 0.01**
How important is practicing a specialization which requires manual work for you?	3.72 (1.61)	**3.63 (1.61)**	**4.06 (1.58)**	**< 0.01**
How important is building a long-term relationship with your patients for you?	3.87 (1.37)	**3.92 (1.35)**	**3.72 (1.44)**	**< 0.01**
How important is it for you that you don’t have to change your place of residence during your further training?	3.95 (1.52)	**4.04 (1.49)**	**3.66 (1.57)**	**< 0.01**
How important is a financial reward for choosing a particular specialization for you?	3.34 (1.40)	3.35 (1.38)	3.29 (1.50)	0.44
How important is the gender distribution of your colleagues (e.g., a predominance of the female or male gender) for choosing a specialization?	2.57 (1.53)	**2.77 (1.54)**	**1.91 (1.29)**	**< 0.01**

* 1 = not important / relevant at all to 6 = very important / relevant; SD: Standard deviation

Correlation analyses with the factors in question were carried out for each of the four different disciplines. Significant correlations were then taken into account in the respective regression analyses.

### Regression analysis

Table 5 shows the results of the binary logistic regression models. The influencing factors that showed a p value of < 0.05 in the forward conditional regression were reported. For the choice of surgery as a specialization, seven factors with a Nagelkerke’s R² value of 0.54 (Hosmer-Lemeshow test *p* < 0.01) were considered in the model. For example, the choice of surgery was significantly associated to work as a senior physician in hospital (ß= 0.60), the importance of a clear structure and regular feedback during specialist training (ß= 0.38), the importance of manual work (ß= 1.44) and less importance concerning long-term relationship with patients (ß= -0.23).

For the desired specialization of anesthesiology, seven factors with a Nagelkerke’s R² value of 0.29 (Hosmer-Lemeshow test *p* = 0.30) were considered in the model. For example, the choice of anesthesiology was significantly associated to work as a senior physician (ß= 1.37) or specialist (ß= 1.52) in hospital and to work 40 h or less, the importance to handle pressure to perform in emergency situation (ß= 0.82), and less importance concerning long-term relationship with patients (ß= -0.41).

For the desired specialization of general practice, eleven factors with a Nagelkerke’s R² value of 0.52 (Hosmer-Lemeshow test *p* = 0.73) were considered in the model. The probability to choose general practice increased significantly to work independent as specialist (OR 9.44, CI 4.64; 19.2) or employed as specialist (OR 6.17, CI 2.65; 14.3) in private practice. Moreover, it was significantly associated with the importance to build a long-term relationship with patients (OR 2.47, CI 1.93; 3.17) and less importance with career opportunities (OR 0.60, 0.49; 0.73).

For the desired specialization of internal medicine, seven factors with a Nagelkerke’s R² value of 0.20 (Hosmer-Lemeshow test *p* = 0.32) were considered in the model. The probability to choose internal medicine increased significantly to be male and to work more than 40 h. Moreover, it was significantly associated with the importance of career opportunities (OR 1.35, CI 1.15; 1.58) and less importance to practice in a specialization which requires manual work (OR 0.61, CI 0.55; 0.69).

**Table 5:** Relevant factors influencing each specialty – presentation of the four regressions models including the regression coefficient (β), odds ratio (OR) and confidence interval (95% CI)

Model 1: General practice	
**Influencing factors**	**β; OR (95% CI); p-value**
Group assignment	1.54; 4.69 (2.75; 7.98); <0.01
Independent specialist in private practice	2.25; 9.44 (4.64; 19.2); <0.01
Employed specialist in private practice	1.82; 6.17 (2.65; 14.3); <0.01
How important is teamwork for you?	-0.54; 0.58 (0.44; 0.78); <0.01
How important are career opportunities for you?	-0.52; 0.60 (0.49; 0.72); <0.01
How important is building a long-term relationship with your patients for you?	0.91; 2.47 (1.93; 3.17); <0.01
How important is place of residence for you when choosing a specialty?	0.30; 1.35 (1.10; 1.66); 0.004
How important are learning opportunities for you?	-0.40; 0.67 (0.52; 0.87); 0.002
How important is the compatibility of family and job for you?	0.34; 1.40 (1.00; 1.97); 0.05
How important is a secure job for you after graduation?	0.53; 1.70 (1.24; 2.31); < 0.01
How important is it for you that you don’t have to change your place of residence during your further training?	0.25; 1.28 (1.05; 1.57); 0.02
**Nagelkerkes R²; (Hosmer-Lemeshow-Test)**	**0.52 (0.73)**
**Model 2: Anesthesiology**	
**Influencing factors**	**β; OR (95% CI); p-value**
Group assignment	0.43; 1.54 (1.02; 2.32); 0.04
Senior physician in hospital	1.37; 3.92 (2.51; 6.12); <0.01
Specialist in hospital	1.52; 4.55 (2.42; 8.56); <0.01
Sex	-0.61; 0.54 (0.36; 0.82); 0.004
40 h or less	0.48; 1.62 (1.09; 2.42); 0.02
How important is building a long-term relationship with your patients for you?	-0.41; 0.66 (0.57; 0.77); <0.01
How well do you think you can handle pressure to perform (e.g. in emergency situations)?	0.82; 2.27 (1.74; 2.97); <0.01
**Nagelkerkes R²; (Hosmer-Lemeshow-Test)**	**0.29 (0.30)**
**Model 3: Surgery**	
**Influencing factors**	**β; OR (95% CI); p-value**
Group assignment	0.50; 1.66 (1.08; 2.56); 0.02
Senior physician in hospital	0.60; 1.82 (1.20; 2.75); 0.01
Semester	-0.08; 0.92 (0.86; 0.98); 0.02
How important is specialist training with a clear structure and regular feedback for you?	0.38; 1.47 (1.09; 1.98); 0.01
How important is it for you that you don’t have to change your place of residence during your further training?	-0.17; 0.84 (0.74; 0.96); 0.01
How important is practicing a specialization which requires manual work for you?	1.44; 4.20 (3.29; 5.37); <0.01
How important is building a long-term relationship with your patients for you?	-0.23; 0.80 (0.68; 0.94); 0.01
**Nagelkerkes R²; (Hosmer-Lemeshow-Test)**	**0.54 (< 0.01)**
**Model 4: Internal medicine**	
**Influencing factors**	**β; OR (95% CI); p-value**
Group assignment	-0.67; 0.52 (0.33;0.81);0.004
Sex	-0.95; 0.39 (0.27; 0.57) ;<0.01
Semester	0.07; 1.07 (1.01; 1.13); 0.02
40 h or less	-0.48; 0.63 (0.44; 0.91); 0.01
How important are career opportunities for you?	0.30; 1.35 (1.15; 1.58); <0.01
How important is practicing a specialization which requires manual work for you?	-0.49; 0.61 (0.55; 0.69); <0.01
How important is direct patient contact in your future job for you?	0.36; 1.43 (1.19; 1.71); <0.01
**Nagelkerkes R²; (Hosmer-Lemeshow-Test)**	**0.20 (0.32)**

## Discussion

The aim of the present study was to explore factors that influence future civilian and military doctors’ choice of specialization. This is of growing importance regarding planning of physicians. A self-created questionnaire was used. For the two topics “student’s skills and abilities” and “general parameters of medical practice” the respectively Cronbach’s alphas were acceptable. Only the variable “importance of practicing a specialization which requires manual work” from the topic “general parameters of medical practice” could not be assigned to a factor. The different factor loadings for the variables of the two topics were good to very good.

The gender and age distribution of the study participants is comparable to those of the general medical students monitoring reports, published every four years [23].

The present survey shows that the quality of specialist training, good team dynamics and job security are key concerns for future physicians. Therefore, these aspects are of great importance for future recruitment of physicians. E.g. specialist training should be as well structured as possible including supervision and well-designed learning targets [24, 25].

The four most frequently chosen specialties were surgery, internal medicine, anesthesiology, and general practice. Interestingly, the factor being a prospective medical officer influenced especially choosing general practice and for civilian students it was internal medicine. It is known that in an armed force the most physicians work as a general practitioner (troop doctor) [26]. If this also influences the career path former studding, should be addressed in future studies.

Moreover, the results of our study are comparable with results from a study published in 2011 [18, 27]. It has shown that the desire to specialize in surgery decreases during the course of studies and increases for general practice [18]. The choice depends on own attitude concerning the specialty would a patient-orientation job, or a job with more manual work or more career option within in the job preferred.

Interestingly, there are significant differences within the two groups of students, that somewhat fit to the future career. For example, prospective medical officers consider communication skills, handling pressure to perform, leadership tasks and to achieving the specialist title as quickly as possible as more relevant than civilian students do. Civilian students consider compatibility of family and job, the place of residence, the importance of building a long-term relationship with patients and the importance not needing to change the place of residence during further training as more relevant than prospective medical officers. Therefore, it can be assumed that different factors should be considered for the allocation of study places. In order to have enough future candidates for all specialties, it seems that different qualifications besides pu-GPA must play a bigger role in order to obtain a place at universities.

A retrospective study with Australian students observed whether there is a relationship between selection criteria of medical students and workplace performance and found moderate associations [28]. Furthermore, a study with German medical students showed that professional and academic pre-qualifications have no advantages for academic success [29]. However, it was found that prior vocational training plays a role as a selection criterion for medical studies [30]. Different approaches to select students for their medical study are discussed [31]. However, the own preferences concerning what kind of future workplace is preferred is not to a sufficient extent of research. As our regression analyses for the different specialty option show, different factors influences whether a student prefer general practice, surgery, anesthesiology or internal medicine. To consider the different preferences might be important for selection medical students. Due to the exploratory design longitudinal research is required whether the own preferences such as the importance of long-term relationship to patients or to prefer to work in hospital or ambulatory setting or practicing in a specialty who required manual work influences the specialty choice. The selection criteria should be supplemented by a large proportion of communication within the process of selection.

### Strengths and weaknesses

The present study is the first which address both, civilian and prospective medical officers in order to generate insights into factors that might determine the future interest in a specialty. Previous studies exclusively address issues of recruitment and retention before and after graduation [8, 32, 33]. The extent to which a selection bias may have existed among the participants in the survey cannot be ruled out with certainty. Additionally, a self-created and non-standardized questionnaire was used for the survey. More research about the psychometric properties on a larger sample would be worthful to examine the reliability and validity of the questionnaire. Due to the study design by using a cross-sectional study design supported by a questionnaire survey, generalizations should only be made with caution.

## Conclusions

Despite close links with the standard civilian German education and training system for physicians, the German Armed Forces have their own recruitment methods, which seem to lead to a different pool of applicants. Differences are particularly evident in the applicants’ choice of specialties in internal medicine and general practice, the latter being more frequently in the military than among civilian students. It seems that different qualifications besides pu-GPA must play a bigger role in order to obtain a place at a medical school to strengthen the recruitment for all different specialties.

## Electronic supplementary material

Below is the link to the electronic supplementary material.

Supplementary Material 1

Supplementary Material 2

Supplementary Material 3

## Data Availability

The datasets used during the current study are available from the corresponding author on reasonable request.

## Abbreviations

pu-GPA: Pre-university grade point average
R²: Total variance
KMO: Kaiser Meyer Olkin
ß: Regression coefficient
OR: Odds Ratio
SD: Standard deviation
95% CI: Confidence interval
