# Supplementary material for: Factors influencing the choice of specialization - a cross-sectional study with civilian medical students and prospective medical officers in Germany
Source: BMC Med Educ. 2024 Oct 17;24:1161. doi: 10.1186/s12909-024-06173-9 (PMC11488281; doi:10.1186/s12909-024-06173-9)
Supplement: Supplementary file 2 — Supplementary Material 2 [file 12909_2024_6173_MOESM2_ESM.pdf]

Additional file: Questionnaire

Are you pursuing a specialty education?

☐yes ☐ yes, but don`t know which one yet ☐no

If yes, what specialty do you plan to pursue after graduation?

☐General medicine
☐Anaesthesiology
☐Occupational medicine
☐Ophthalmology
☐General and visceral surgery
☐Trauma surgery and orthopedics
☐Cardiac, thoracic and vascular surgery
☐Plastic surgery
☐Dermatology and venereology
☐Gynecology
☐Otolaryngology
☐Hygiene
☐Internal medicine
☐Endocrinology and diabetology
☐Gastroenterology
☐Hematology and oncology
☐Cardiology
☐Angiology
☐Pneumology
☐Clinical chemistry and laboratory medicine
☐Microbiology
☐Oral and maxillofacial surgery
☐Neurosurgery
☐Neurology
☐Nuclear medicine
☐Public health
☐Pathology
☐Pharmacology, toxicology
☐Psychiaty
☐Radiology
☐Transfusion medicine
☐ Urology
☐ Other, and that is:______

Please rate from 1 not important at all/ relevant = to 6= very important/ relevant:

How confident do you feel about examining the musculoskeletal system?
☐ ☐ ☐ ☐ ☐ ☐

How would you rate the amount of your curricular training on musculoskeletal examination?
☐ ☐ ☐ ☐ ☐ ☐
How competent do you consider yourself in terms of your communication skills?
☐ ☐ ☐ ☐ ☐ ☐
How important is a financial reward for choosing a particular specialization for you?
☐ ☐ ☐ ☐ ☐ ☐
How important is a secure job for you after graduation?
☐ ☐ ☐ ☐ ☐ ☐
How important is the compatibility of family and job for you?
☐ ☐ ☐ ☐ ☐ ☐
How important are learning opportunities for you?
☐ ☐ ☐ ☐ ☐ ☐
How important is specialist training with a clear structure and regular feedback for you?
☐ ☐ ☐ ☐ ☐ ☐
How important is it for you to achieve your specialist title as quickly as possible?
☐ ☐ ☐ ☐ ☐ ☐
How important are career opportunities for you?
☐ ☐ ☐ ☐ ☐ ☐
How important is a place of residence to you when choosing a specialty?
☐ ☐ ☐ ☐ ☐ ☐
How important is the gender distribution of your colleagues (e.g., a predominance of the female or male gender) for you when choosing a specialty?
☐ ☐ ☐ ☐ ☐ ☐
How important is it for you that you don’t have to change your place of residence during your further training?
☐ ☐ ☐ ☐ ☐ ☐
How important is an option of changing specialization if necessary for you?
☐ ☐ ☐ ☐ ☐ ☐
How important is practicing a specialization which requires manual work for you?
☐ ☐ ☐ ☐ ☐ ☐
How important is teamwork for you?
☐ ☐ ☐ ☐ ☐ ☐
How important is direct patient contact in your future job for you?
☐ ☐ ☐ ☐ ☐ ☐
How important is building a long-term relationship with your patients for you?
☐ ☐ ☐ ☐ ☐ ☐
How well do you feel qualified for leadership tasks (e.g. leading a team)?
☐ ☐ ☐ ☐ ☐ ☐
How well do you think you can handle pressure to perform (e.g., in emergency situations)?
☐ ☐ ☐ ☐ ☐ ☐

Which professional position do you want to achieve?
☐Head physician in hospital
☐Senior physician in hospital
☐Specialist in hospital

☐Independent specialist in private practice
☐Specialist in employed relationship
☐Non-medical work (journalism, consulting)
☐Lab work, research
☐Official activity, administration
☐Others and that is: ____________

Where would you like to work later?
☐ Rural area in Germany
☐ Town in Germany
☐ Abroad

Age (in years): _____

Sex: ☐male ☐ female ☐divers

Do you live in a committed partnership? (e.g. marriage) ☐ Yes ☐ No

Do you have children? ☐ Yes ☐ No

If yes, how many children?

Semester: ☐1 ☐2 ☐3 ☐4 ☐5 ☐6 ☐7 ☐8 ☐9 ☐10 ☐11 ☐12

University: ☐Aachen ☐Augsburg ☐Berlin ☐Bochum ☐Bonn ☐Dresden ☐ Duisburg-Essen ☐Düsseldorf ☐Frankfurt/Main ☐Freiburg ☐Gießen ☐Göttingen ☐Greifswald ☐Halle ☐Hamburg ☐Hannover ☐Heidelberg ☐Jena ☐Kiel ☐Köln ☐Leipzig ☐Lübeck ☐Magdeburg ☐Mainz ☐Mannheim ☐Marburg ☐München ☐Münster ☐Erlangen-Nürnberg ☐Oldenburg ☐Regensburg ☐Rostock ☐ Saarland/ Homburg ☐Tübingen ☐Ulm ☐Witten/ Herdecke ☐Würzburg

Access to study:
☐ Abitur grade
☐ University selection procedures
☐ Waiting period
☐ German Armed Forces
☐ Other and that is: ________

District where you grew up mostly: ______________________

Soldier? ☐ Yes ☐ No

Thank you
