# Supplementary material for: Factors influencing the choice of specialization - a cross-sectional study with civilian medical students and prospective medical officers in Germany
Source: BMC Med Educ. 2024 Oct 17;24:1161. doi: 10.1186/s12909-024-06173-9 (PMC11488281; doi:10.1186/s12909-024-06173-9)
Supplement: Supplementary file 3 — Supplementary Material 3 [file 12909_2024_6173_MOESM3_ESM.pdf]

Additional files: Tables

Additional file Table 1: Exploratory factor analysis for the topic: "students' skills and abilities"

Variable overview	Factor loading*	
	Factor 1	Factor 2
How competent do you consider yourself in terms of your communication skills?	0.674	0.152
How well do you think you can handle pressure to perform (e.g. in emergency situations)?	0.791	0.106
How well qualified do you feel for leadership tasks (e.g. leading a team)?	0.822	0.095
How confident do you feel about examining the musculoskeletal system?	0.266	0.782
How would you rate the extent of your curricular training on musculoskeletal examination?	0.021	0.876

Additional file Table 2: Exploratory factor analysis for the topic: "general parameters of medical practice"

Variable overview	Factor loading*				
	Factor 1	Factor 2	Factor 3	Factor 4	Factor 5
How important is specialist training with a clear structure and regular feedback for you?	0.152	0.054	0.715	0.065	-0.017
How important is teamwork for you?	0.031	-0.140	0.553	0.361	0.168
How important is a secure job for you after graduation?	0.472	0.216	0.391	-0.048	-0.219
How important are learning opportunities for you?	-0.161	0.173	0.667	-0.020	0.055
How important is direct patient contact in your future job for you?	0.028	-0.063	0.175	0.835	0.003
How important is the compatibility of family and job for you?	0.685	-0.119	0.103	0.171	0.102
How important are career opportunities for you?	-0.232	0.764	0.193	-0.116	0.076
How important is it for you to achieve your specialist title as quickly as possible?	0.123	0.693	0.146	0.102	-0.219
How important is place of residence for you when choosing a specialty?	0.696	0.129	-0.073	0.028	0.214
How important is an option of changing specialization if necessary for you?	0.243	-0.086	0.265	-0.086	0.603
How important is practicing a specialization which requires manual work for you?	-0.343	0.288	0.307	-0.062	0.208
How important is building a long-term relationship with your patients for you?	0.203	-0.067	-0.044	0.820	0.095
How important is it for you that you don't have to change your place of residence during your further training?	0.729	0.035	-0.023	0.074	0.083
How important is a financial reward for choosing a particular specialization for you?	0.224	0.682	-0.150	-0.206	0.157
How important is the gender distribution of your colleagues (e.g., a predominance of the female or male gender) for choosing a specialization?	0.073	0.081	-0.062	0.189	0.757
